# Effects of Soil Data and Simulation Unit Resolution on Quantifying Changes of Soil Organic Carbon at Regional Scale with a Biogeochemical Process Model

**DOI:** 10.1371/journal.pone.0088622

**Published:** 2014-02-11

**Authors:** Liming Zhang, Dongsheng Yu, Xuezheng Shi, Shengxiang Xu, Shihe Xing, Yongcong Zhao

**Affiliations:** 1 College of Resource and Environment, Fujian Agriculture and Forestry University, Fuzhou, China; 2 State Key Laboratory of Soil and Sustainable Agriculture, Institute of Soil Science, Chinese Academy of Sciences, Nanjing, China; Catalan Institute for Water Research (ICRA), Spain

## Abstract

Soil organic carbon (SOC) models were often applied to regions with high heterogeneity, but limited spatially differentiated soil information and simulation unit resolution. This study, carried out in the Tai-Lake region of China, defined the uncertainty derived from application of the DeNitrification-DeComposition (DNDC) biogeochemical model in an area with heterogeneous soil properties and different simulation units. Three different resolution soil attribute databases, a polygonal capture of mapping units at 1∶50,000 (P5), a county-based database of 1∶50,000 (C5) and county-based database of 1∶14,000,000 (C14), were used as inputs for regional DNDC simulation. The P5 and C5 databases were combined with the 1∶50,000 digital soil map, which is the most detailed soil database for the Tai-Lake region. The C14 database was combined with 1∶14,000,000 digital soil map, which is a coarse database and is often used for modeling at a national or regional scale in China. The soil polygons of P5 database and county boundaries of C5 and C14 databases were used as basic simulation units. Results project that from 1982 to 2000, total SOC change in the top layer (0–30 cm) of the 2.3 M ha of paddy soil in the Tai-Lake region was +1.48 Tg C, −3.99 Tg C and −15.38 Tg C based on P5, C5 and C14 databases, respectively. With the total SOC change as modeled with P5 inputs as the baseline, which is the advantages of using detailed, polygon-based soil dataset, the relative deviation of C5 and C14 were 368% and 1126%, respectively. The comparison illustrates that DNDC simulation is strongly influenced by choice of fundamental geographic resolution as well as input soil attribute detail. The results also indicate that improving the framework of DNDC is essential in creating accurate models of the soil carbon cycle.

## Introduction

An estimated 1500 Pg of C is held in the form of soil organic carbon (SOC), representing 2/3 of the global terrestrial organic carbon pool [1]–[3]. SOC plays a vital role in the global carbon cycle, where a slight alteration of the soil carbon pool can cause profound changes in atmospheric CO_2_ concentrations. Agro-ecosystems, accounting for 10% of the total terrestrial area, are one of the most sensitive terrestrial ecosystems subject to heavy human activity [3]. Increasing agricultural soil C sequestration is recognized as one strategy for achieving food security and improving soil quality.

Paddy soil is a major cultivated soil in China, and a unique type of anthropogenic soil recognized by Chinese Soil Taxonomy [3]–[5]. The total area of paddy soils is 45.7 M ha, which accounts for 34% of the total cultivated land in China [6]. This area also accounts for 22% of the total waterlogged farming area worldwide and produces about 44% of all grain in China [4]. Therefore, accurate estimation of paddy soil SOC change in China is vitally important for a comprehensive understanding of SOC dynamics and agro-ecosystem sustainability.

Recently, scientists have applied modeling to estimate SOC change in cropping systems [7]–[14]. The DeNitrification-DeComposition (DNDC) model, developed by Li et al. [15], [16], is a process-based model focused on agrosystem carbon and nitrogen cycling and has been widely used for regional studies in the USA [17], China [11], India [18] and Europe [19]. Recently the DNDC model was determined to be one of the well performing models based on seven long-term experiments selected by the Global Change and Terrestrial Ecosystems Soil Organic Matter Network (GCTE SOMNET), which evaluated model performance using three different land uses, a range of climatic conditions within the temperate region, and different treatments [11], [14].

In China, scientists have studied SOC change using the DNDC model for many years. At the regional scale, Tang et al. [11] simulated SOC changes for cropland in China for 1998 using the DNDC model, and they found that SOC would be lost at a rate of 78.89 Tg C year^−1^. Zhang et al. [20] linked the DNDC model and 1: 14,000,000 soil database to estimate SOC stock changes for the year 2000 in Northwest China, revealing a decline in SOC stock. At the field scale, Wang et al. [21] tested DNDC uncertainty based on six long-term (10–20 year) SOC datasets from the Northeast, North, Northwest, Central South, East, and Southwest China. Results from the six validation tests supported the previous conclusions that the DNDC model was capable of quantifying SOC change in the agroecosystems across the entire area of China.

To date, the county boundary was used as the basic simulation unit in most DNDC simulations conducted at regional scale [11], [20]. As a result, these simulations are often subject to great uncertainties since the soil property data were averaged for the area, which greatly ignore the impacts of soil heterogeneity therein [18], [22]. Moreover, many researchers used coarse soil attribute data obtained from the books such as Soil in China (Vol. 1–6) and 1: 14,000,000 soil maps at national or a regional scale in China [11], [20]. However, studies have already pointed out that the effect of soil heterogeneity on SOC change estimation is a major source of uncertainty when using the DNDC model at the regional scale [18], [22], [23].

This study, which was carried out in the rice-dominated Tai-Lake Region of China, provides a chance to test the uncertainty of the DNDC model caused by different precisions of soil data and basic simulation unit. The goals of this study were to: (1) compare SOC changes modeled with different resolutions of soil databases and varied basic simulation units, (2) assess the uncertainty derived from these soil databases with different resolutions and basic simulation units, and (3) give some suggestions for improving the performance of the biogeochemical DNDC model applied at the regional scale.

## Materials and Methods

### Study area

**Figure 1:**
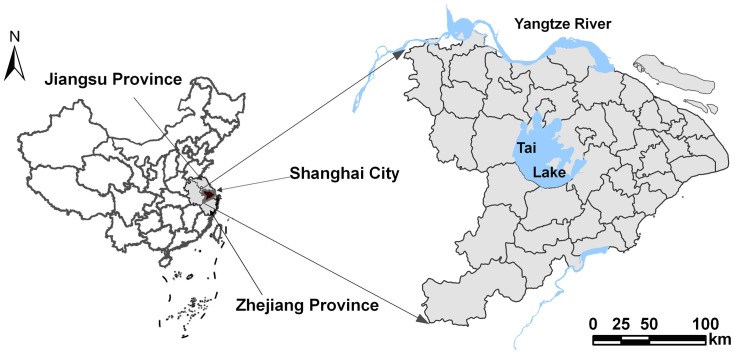
Geographical location of the study area in China.

The Tai-Lake region (118°50′-121°54′E, 29°56′-32°16′N), an area of intensive rice cultivation, is located in the middle and lower reaches of the Yangtze River paddy soil region of China. The region includes the entire Shanghai City administrative area and a part of Jiangsu and Zhejiang provinces, and covers a total area of 36,500 km^2^ (**Fig. 1**) [4]. The Tai-Lake region mainly consists of plains formed on deltas with numerous rivers and lakes. The climate is warm and moist with abundant sunshine and a long growing season. Annual rainfall is 1,100–1,400 mm, with a mean temperature of 16°C, and average annual sunshine of 1,870–2,225 hours. The frost-free period is over 230 days. The study area is one of the oldest agricultural regions in China, with a long history of rice cultivation spanning several centuries. Most cropland in the region is managed as a rice and winter wheat rotation. Rice is planted in June and harvested in October and wheat is planted in November and harvested in May [24].

Approximately 66% of the total land area is covered with paddy soils [24]. Paddy soils in the Tai Lake area are derived mostly from loess, alluvium, and lacustrine deposits, and are classified into 6 soil subgroups according to the Genetic Soil Classification of China (GSCC) system which are represented in the 1∶50,000 digital soil map (**Table 1**). As map scale decreased, the soil subgroups of submergenic, bleached, percogenic and degleyed on the 1∶50,000 soil map was eliminated and emerged into the soil subgroups of degleyed and hydromorphic in the 1∶14,000,000 soil map. Therefore, those were only two paddy soil subgroups of degleyed and hydromorphic in the 1∶14,000,000 soil map. The GSCC nomenclature as well as the subgroup's reference name in US Soil Taxonomy (ST) include; Hydromorphic (Typic Epiaquepts), Submergenic (Typic Endoaquepts), Bleached (Typic Epiaquepts), Gleyed (Typic Endoaquepts), Percogenic (Typic Epiaquepts), and Degleyed (Typic Endoaquepts) [25], [26].

**Table 1:** The subgroups of paddy soil in the Tai-Lake region, China.

Subgroups	Horizonation*	Descriptions
Bleached	A-P-E-C	Mainly distributed in foothills, usually no underground water, impervious layer at 60 cm depth, soil reaction close to neutral or slightly acid.
Gleyed	Aa-Ap-G-C	Mainly distributed in depressional areas, high underground water level, poorly drained, distinct gleyization, soil reaction was slightly acid.
Percogenic	Aa-Ap-C	Mainly distributed on gentle hill slopes, no underground water, associated with rain-fed paddy fields, soil reaction was neutral to slightly acid.
Degleyed	Aa-Ap-Gw-G	Same distribution area as Gleyed paddy soils, after man-made drainage the underground water level decreases leading to degley processes, soil reaction was slightly acid.
Submergenic	A-Ap-P-C	Mainly distributed in alluvial plain or low flat ground, moderate drainage, underground water level was below 60 cm, soil reaction was neutral.
Hydromophic	Aa-Ap-P-W-G-C	Mainly distributed in floodplain, long cultivation history, well-drained, underground water level was below 90 cm, soil reaction was neutral.

^*^According to GSCC (Genetic Soil Classification of China), Aa means arable layer, Ap plow pan, C undeveloped parent material, Ds fragmental deposit horizon, E bleached horizon, G gley horizon, Gw degley horizon, P percogenic horizon, W waterlogogenic horizon.

### Description of the DNDC model

The DNDC model (Version 9.1) is a process-based soil biogeochemical research tool that was developed to estimate the impact of management strategies on the fate of nitrogen (N) and carbon (C) in agroecosystems. It integrates crop growth and soil biogeochemical processes on a daily time step and simulates N and C cycles in plant-soil systems.

The model contains six interacting sub-models which describe the generation, decomposition, and transformation of organic matter, and outputs the dynamic components of SOC and greenhouse gas fluxes. The six sub-models include: 1) a soil climate component which use soil physical properties, air temperature, and precipitation data to calculate soil temperature, moisture, and redox potential (Eh) profiles and soil water fluxes through time. The results of the calculation are then fed to the other sub-models; 2) a nitrification component; 3) a denitrification module, which calculates hourly denitrification rates and N_2_O, NO, and N_2_ production during periods when the soil Eh decreases due to rainfall, irrigation, flooding, or soil freezing; 4) simulation of SOC decomposition and CO_2_ production through soil microbial respiration; 5) a plant growth component, which calculates daily root respiration, water, and N uptake by plants, and plant growth; and 6) a fermentation module, which calculates daily methane (CH_4_) production and oxidation. The DNDC model can simulate C and N biogeochemical cycles in paddy rice ecosystems, as the model has been modified by adding a series of anaerobic processes [15], [16], [22], [23], [27], [28], [29], [30].

At present, the DNDC model has been utilized by scientists in many countries, for example, the model is applied to simulate the carbon cycle in paddy field in Italy, China and Germany, in wheat fields in Canada, and it has been used to simulate the dynamics of soil organic matter in a 100 year experimental field in Rothamsted Experimental Station in England [14], [31]. At the international conference on global change in Asia-Pacific areas in 2000, the DNDC model was recommended as the primary method for SOC studies in the in the Asia-Pacific region [31].

### Database development

A major challenge for using an ecosystem model at regional scale is to assemble adequate datasets required to initialize and run the model. We examined the influence of database choices by executing simulation runs with different input sets using individual or combinations of databases. The geographic resolution or fundamental simulation unit could be represented by any of three assessment unit format datasets, polygon-based database of 1∶50,000 (P5), county-based database of 1∶50,000 (C5), and county-based database of 1∶14,000,000 (C14). The three soil datasets covered 37 counties in Tai-Lake region.

The polygon-based database of 1∶50,000 (P5) was linked a digital soil map (1∶50,000), the most detailed of the three databases, in the Tai-Lake region contains 52,034 paddy soil polygons (**Table 2**). The polygons were derived from 1,107 soil profiles extracted from the latest national soil map (1∶50,000), the Second National Soil Survey of China in the 1980s-1990s, with attribute assignment using the Pedological Knowledge Based (PKB) method based on GSCC [32]. The 1∶50,000 digital soil database consists of many soil attributes, such as soil name, horizon thickness, bulk density, organic carbon content, clay content, pH, etc.

**Table 2:** Characteristics of different resolution soil attribute databases of paddy soils in GSCC in the Tai-Lake region, China.

Soil database	Map scale	Source of soil maps	Source of soil data	Basic map units	Number of soil profiles	Number of polygons	Simulation unit
P5	1∶50,000	Soil Survey Office of County in Jiangsu Province, Zhejiang Province and Shanghai City	Soil Series of County in Jiangsu Province, Zhejiang Province and Shanghai City	Soil Species	1,107	52,034	polygon
C5	1∶50,000	Soil Survey Office of County in Jiangsu Province, Zhejiang Province and Shanghai City	Soil Series of County in Jiangsu Province, Zhejiang Province and Shanghai City	Soil Species	1,107	52,034	county
C14	1∶14,000,000	Institute of Soil Science, Chinese Academy of Sciences	Soil Series of China	Subgroups	49	8	county

**Figure 2:**
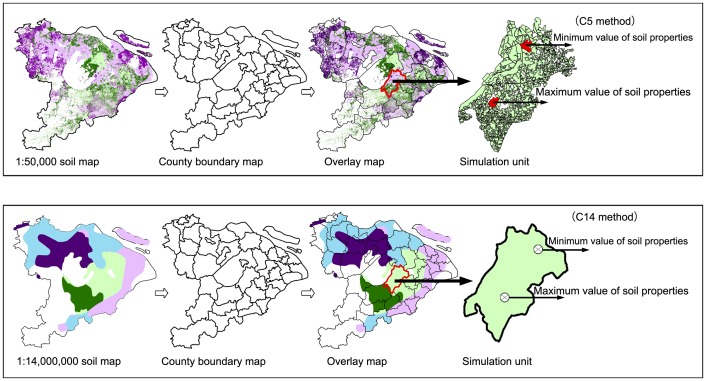
Description of C5 and C14 methods in the Tai-Lake region of China.

Soil parameters in C5 were derived from the 1∶50,000 digital soil map (**Fig. 2** **and** **Table 2**). However the attributes for C14 were derived from different sources than C5, primarily the 1∶14,000,000 national soil map [33], [34] (**Fig. 2**). C14 was widely used when the DNDC model was applied to national or regional scale in China [11], [20]. The C14 in the Tai-Lake region contained 8 polygons of paddy soils representing 49 paddy soil profiles, and was also compiled via the Pedological Knowledge Based (PKB) method based on GSCC [32].

The C5 and C14 were built from the default method developed for DNDC, in which the maximum and minimum values of soil texture, pH, bulk density, and organic carbon content were recorded for each county (**Fig. 2**). So, the DNDC modeling of C5 and C14 methods conducted have used counties as the basic simulation unit in the Tai-Lake region (**Fig. 2**). After regional runs with C5 and C14 database, the DNDC model produced two SOC change (0–30 cm) resulting from two runs with the maximum and minimum soil values in each county. In this paper we present the mean results (average of maximum and minimum estimates) [11]. The DNDC modeling of P5 method conducted has used polygon as the basic simulation unit in the Tai-Lake region (**Table 2**). Therefore, the DNDC model runs with P5 database produced a single annual SOC change (0–30 cm) for each polygon. The total SOC change of each county in the P5 was calculated by summing the SOC change of all polygons in a county. For a more complete description of P5 method see Zhang et al [35], [36] and Xu et al [37].

For comparison in this study, both the polygon-based (P5) and county-based (C5 and C14) soil databases in the Tai-Lake region were run concurrently so the DNDC model could generalize regional SOC change from 1982 to 2000. The results simulated by DNDC with the two types of databases were compared to assess the advantages of using detailed, polygon-based 1∶50,000 soil dataset (P5) [35], [36], [38], [39].

In this study, the crop dataset included physiological data for summer rice and winter wheat in the Tai-Lake region. The crop parameters were obtained from thorough testing with that reflected the typical conditions of Tai-Lake region, which were founded on a wide range of information form Chinese literature published during the past decade and a publication of Gou et al [40], [41].

**Figure 3:**
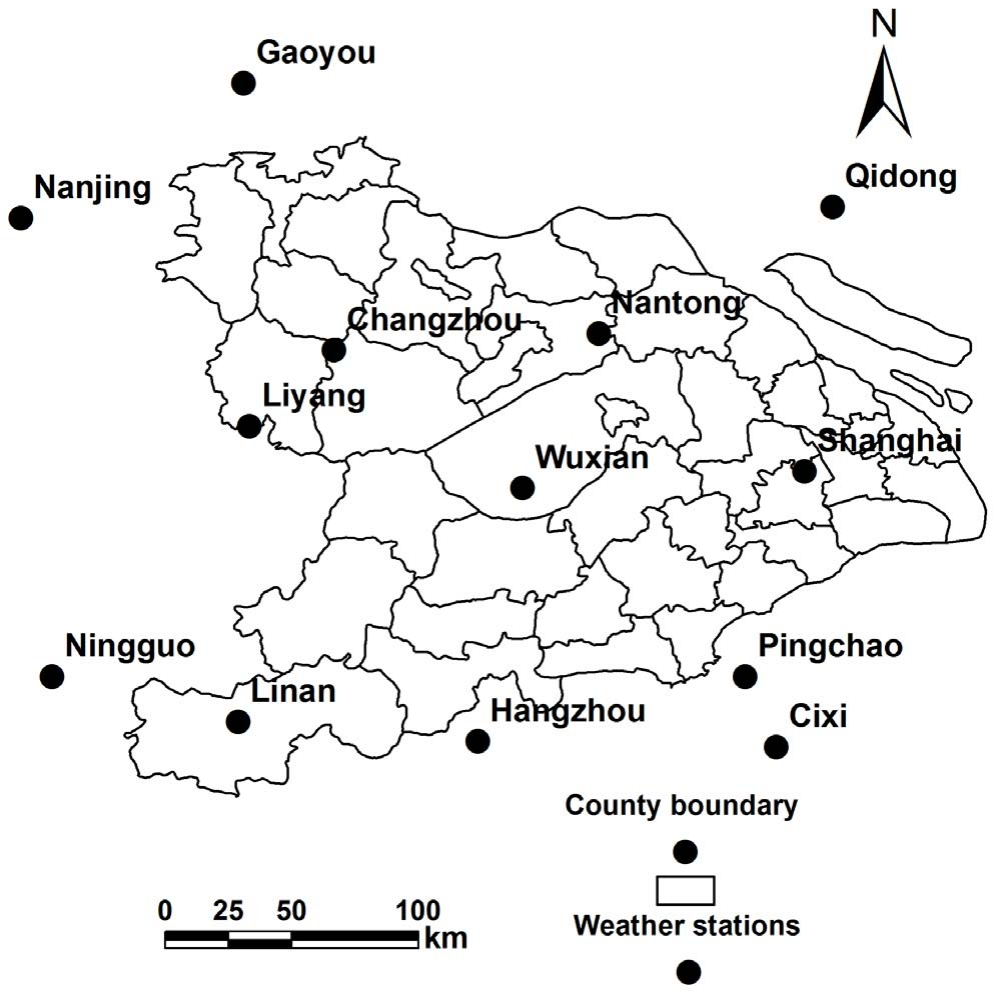
Geographical location of weather stations across or near the Tai-Lake region, China.

Daily meteorological data (precipitation, maximum and minimum air temperature) for 1982–2000 from 13 weather stations across and near the Tai-Lake region were acquired from the National Meteorological Information Center, China Meteorological Administration (**Fig. 3**) [42]. Each county in the simulation was assigned to the nearest weather station [11], [20], [31].

The agricultural management dataset included sowing acreage, nitrogen fertilizer application rates, livestock, planting and harvest dates, and agricultural population at the county level from 1982 to 2000 in three resolution databases. The crop management practices of different counties were almost the same because the Tai-Lake region was a plain in topography. The main measures of farming management in the study area included: (1) fertilizer application: nitrogen synthetic fertilizer was applied for 6 times in the basal, tillering and heading stage for rice, and in the basal, jointing and heading stage for wheat; and organic manure (20% of livestock wastes and 10% of human wastes) was applied twice as base fertilizer for rice and wheat at the rates calculated based on the local livestock numbers (866, 44, 95, and 23 kg C head^−1^ yr^−1^ for cattle, sheep, swine and human, respectively); and N concentration in rainfall was 2.07 ppm; (2) crop residue management: 15% of aboveground crop residue was returned to the soil; (3) water management: one time of midseason and 5 time of shallow flooding (from June 17 to July 23, from July 28 to August 12, from August 24 to September 11, from September 18 to September 25, and from September 27 to October 2, respectively) were applied at summer rice; (4) tillage: twice at the 20 cm tilling depth for rice and 10 cm for wheat on the planting dates before 1990; and no-till applied for wheat after 1990; (5) growing period: rice is planted in June and harvested in October and wheat is planted in November and harvested in May; (6) optimum yield: rice is 7500 kg dry matter ha^−1^ and wheat is 3750 kg dry matter ha^−1^ [11], [14], [24], [35], [41], [43]. All simulation methods within a certain county have the same feature input value such as crops, agricultural management, and climate, except soil feature [38], [39].

### Evaluation of simulation accuracy in three resolution databases

In order to evaluate the accuracy in three resolution databases, the simulated results of DNDC model were tested against measured data from paddy soils of the Tai-Lake region, which is the same area examined here.

From the perspective of previous studies, most dynamic models were only tested or validated with static long-term field-scale observations due to a lack of available soil data with temporal and spatial variation. Since these models have not yet been validated by regional scale data, uncertainty concerning their accuracy exists when they were applied to larger area dynamic SOC simulation [3].This study compared simulation results with the spatial distribution of SOC measurements from 1033 paddy soil sampling sites acquired in 2000, to validate and assess model performance in different simulation methods (P5, C5, and C14). The bias in the total difference between simulation and measurement were determined by calculating the correlation coefficient (r), the relative error (E), the mean absolute error (MAE) and the root mean square error (RMSE), as follows: [8], [44]. 
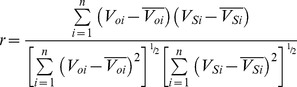
(1)

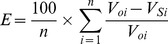
(2)

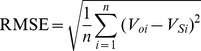
(3)

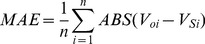
(4)Where *V_oi_* are the observed values, 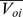 is the mean of the observed data, 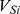 is the simulated value, 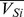 is the mean of the simulated value, 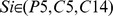, and n is the number in the sequence of the data pairs. If E is less than 5% or between 5% and 10%, the simulation is satisfactory or acceptable, respectively; otherwise, it is unacceptable [44]. The greater r value is and the smaller RMSE or MAE value is, the greater prediction accuracy is. Conversely, a lower r value and more elevated RMSE or MAE value, the lower prediction accuracy is.

### Data comparison and analysis

SOC change as quantified by DNDC modeling with the P5 assessment unit data set are recognized as a benchmark for comparison with the results of the DNDC model runs with the other two assessment unit data sets as input. The P5 are thought theoretically to be more accurate than the C5 and C14 because of their relative greater detail and accuracy [35], [36], [38], [39]. Relative variation of an index value (VIV, %) of C5 and C14 methods is calculated as the formula (1). The index values (IV) were quantified from the P5 data set (IV_P5_) and other data sets (IV_Ci_) to support data set comparison [23], [38], [39].
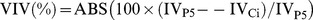
(5)Where ABS is the absolute value function, IV_P5_ is the total SOC change with P5, and IV_Ci_ is the total SOC change produced by C5 (or C14).

Previous results from the sensitivity tests of the DNDC model indicated that the spatial heterogeneity of soil properties (e.g. texture, SOC content, bulk density, and pH) are the major sources of uncertainty for simulating SOC changes under specific management conditions at regional scale [18], [20], [22], [23]. In order to test the most sensitive soil properties factor, the correlation of soil properties and average annual SOC changes were determined by step-wise regression analysis by using SPSS statistical software [37], [45]. The step-wise regression is useful in checking how entering each variable affects the overall regression model, which begins by entering the variable with the largest partial statistic and checking the importance of the coefficient of the variable [45], [46]. This method keeps adding more variables, each time recalculating the coefficients. During the incorporation of a variable into the model, the partial statistic of the already entered variable changes and might cause it to be unimportant. The operation stops when the model has incorporated the variables with the most significant contribution and discarded the least significant ones [47].

## Results and Discussion

### Difference of simulation accuracy in three resolution databases

**Figure 4:**
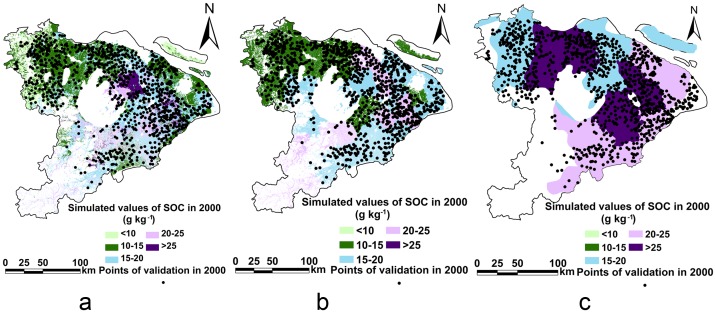
Spatial distribution of validation points and simulated SOC values from different simulation methods for the Tai-Lake region for 2000 (a: P5, b: C5, and c: C14).

**Figure 5:**
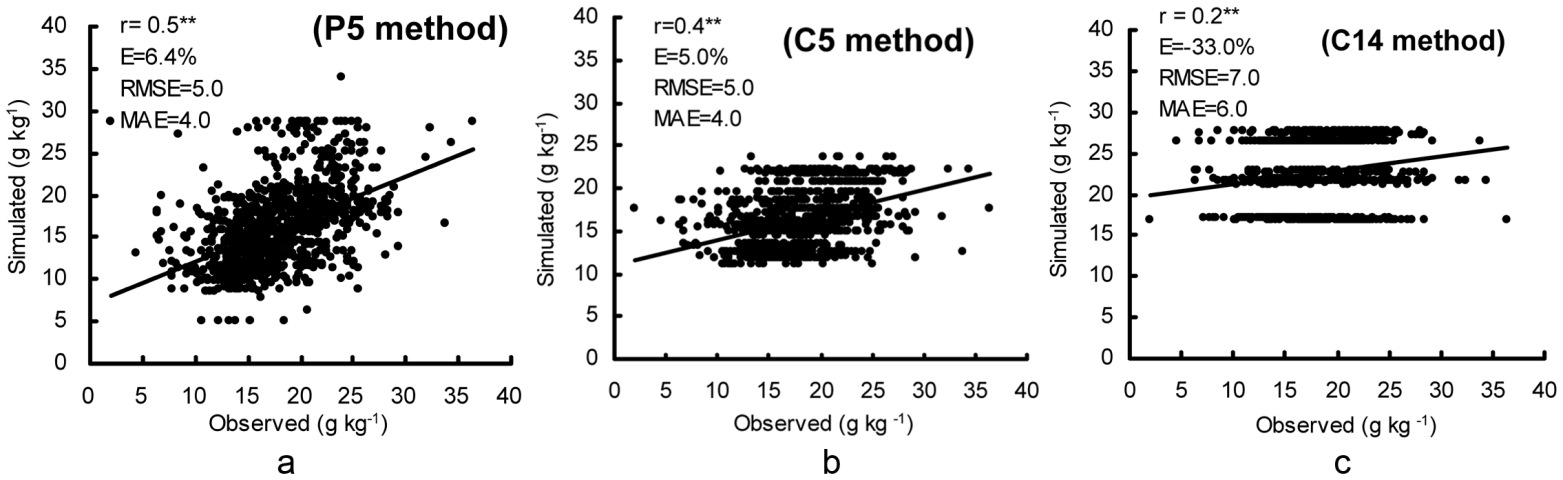
Comparison between simulated and observed SOC values from different simulation methods of the Tai-Lake region for 2000 (a: P5, b: C5, and c: C14).

Three maps of average SOC content for paddy soils at surface layers (0–15 cm) in the study area in 2000 were constructed on the basis of simulated data in different simulation methods (P5, C5, and C14) (**Fig. 4**). Also, corresponding SOC validation points were constructed from measurements of the surface layer (0–15 cm) of 1033 paddy soil samples taken in the study area in 2000. Fig. 4 demonstrates that the observed SOC in 2000 varied from 1.9 g kg^−1^ to 36 g kg^−1^. By comparison, Fig. 4 also illustrates that simulated SOC in 2000 varied from 5.1 g kg^−1^ to 34 g kg^−1^ in P5, from 11 g kg^−1^ to 24 g kg^−1^ in C5, and from 17 g kg^−1^ to 28 g kg^−1^ in C14; where 99.6%, 84.1% and 57.1% of simulated paddy soil samples in P5, C5 and C14 were within the ranges produced by the observed SOC data. Furthermore, the relative errors (E) of P5 and C5 were 6.4% and 5.0%, respectively; and within the range of 5%–10%, demonstrating that the DNDC model in P5 and C5 were acceptable for modeling SOC of paddy soils in the Tai-Lake region according to the evaluation criteria described earlier (**Fig. 5a and b**) [8], [44]. Moreover, the small values of MAE (4.0 g kg^−1^) and RMSE (5.0 g kg^−1^) in P5 and C5 also indicated that the modeled results were encouragingly consistent with observations in the Tai-Lake region (**Fig. 5a and b**). However, the E, MAE and RMSE of C14 reached −33%, 6.0 g kg^−1^ and 7.0 g kg^−1^, respectively, suggesting that the simulated results of C14 were not suitable for simulating paddy soils in the Tai-Lake region (**Fig. 5c**).

Overall, though the values of E, MAE and RMSE between P5 and C5 had no significant differences, P5 was recognized better due to high correlation coefficient (0.5) and accurate simulation range (99.6%) (**Fig. 5a and b**). Furthermore, the simulation of P5 can differentiate the difference of paddy soil type within a county. Some studies showed that SOC content spatial variability was correlated with soil type spatial variability(**Fig. 5a**) [32], [48], [49]. Compared to the SOC validation of DNDC model in cropland by other scientists, accurate simulation of P5 (r = 0.50^**^; E = 6.4%; MAE = 4.0 g kg^−1^; RMSE = 5.0 g kg^−1^ and n = 1033) and C5 (r = 0.40^**^; E = 5.0%; MAE = 4.0 g kg^−1^; RMSE = 5.0 g kg^−1^ and n = 1033) are higher than those of Liu et al [50] (r = 0.25–0.66 and n = 68), Liu et al [51] (E = 27.6% and n = 49), and Xu et al [52] (r = 0.22^**^; RMSE = 4.4 g kg^−1^ and n = 243); and are almost similar to that of the Xu et al [52] (r = 0.52^**^; RMSE = 4.1 g kg^−1^ and n = 1385). However, the SOC accurate simulation of DNDC model in P5, C5 and C14 are lower than those of Studdert et al [53] (r = 0.73^**^ and n = 286) by using the RothC model and Yu et al [54] (r = 0.98^**^ and n = 349) by using the Agro-C model. Therefore, the results mentioned above suggest that modification of the DNDC model is necessary to better simulate SOC change from cropping systems. With continued modification, DNDC model could become a powerful tool for estimating SOC change at regional and national scales.

### Variation of soil properties derived as input for DNDC modeling in three resolution databases in Tai-Lake region

Results of the contribution of soil properties to the variability of average annual SOC change are given in Table 3. All variables (i.e., initial SOC content, pH, bulk density, and clay content) were included in the step-wise regression analysis. For the P5 and C5 resolution databases, initial SOC content accounted for 77.8%–88.1% of the difference of average annual SOC change for paddy soils from 1982 to 2000, while other soil parameters only accounted for less than 6.6% of the difference. For the C14 resolution database, initial SOC content accounted for 18.5% of the difference of average annual SOC change for paddy soils from 1982 to 2000, and soil pH accounted for 75.7% of the difference. Therefore, it could be inferred that the differences in SOC change modeled with the three resolution databases were primarily due to the differences in initial SOC content and pH.

**Table 3:** Soil properties at three resolution soil attribute databases contributing to the variability of average annual SOC change in Tai-Lake region paddy soils from 1982 to 2000.

Soil database	Number of simulation units	△R^2a^				Adjusted R^2^
		Initial SOC (g kg^−1^)	Clay(%)	pH	Bulk density (g cm^−3^)	
P5	52,034	0.778***	0.066***	0.009***	0.025***	0.878***
C5	37	0.881***			0.062***	0.939***
C14	37	0.185***		0.757***		0.938***

^***^significant at 0.001 probability levels, respectively.

a The change in the R^2^ statistic is produced by adding a soil property into stepwise multiple regressions.

**Table 4** shows the initial SOC content (0–5 cm), clay content (0–10 cm), pH (0–10 cm), and bulk density (0–10 cm) derived as input for DNDC modeling, from P5, C5 and C14 for the Tai-Lake region. As for the entire Tai-Lake region, the average initial SOC values sourced from P5 was lower than that from C5 and C14. Another difference is that the average values of clay content and pH sourced from C14 were also higher than those from P5 and C5. The average bulk density sourced from C5 was higher than that from P5 and C14.

**Table 4:** Statistics for soil properties derived as input for DNDC modeling in different counties, from P5, C5 and C14 for the Tai-Lake region.

County	P5				C5								C14							
	SOC	Clay	BD	pH	SOC		Clay		BD		pH		SOC		Clay		BD		pH	
	-----WA------				Range	Ave	Range	Ave	Range	Ave	Range	Ave	Range	Ave	Range	Ave	Range	Ave	Range	Ave
Zhangjiagang	14	28	1.22	7.7	10–17	14	5–32	19	1.16–1.33	1.25	7.4–8.0	7.7	12–21	17	24–31	28	1.19–1.23	1.21	6.0–7.4	6.7
Changshu	17	25	1.23	7.0	9–38	24	9–34	22	1.05–1.46	1.26	5.5–8.1	6.8	12–21	17	24–31	28	1.19–1.23	1.21	6.0–7.4	6.7
Taicnang	14	33	1.20	7.7	9–20	15	23–42	33	1.11–1.38	1.25	7.4–8.6	8.0	11–31	21	18–48	33	1.12–1.27	1.20	5.5–7.4	6.5
Kunshan	19	35	1.15	7.1	11–34	18	22–44	33	0.94–1.40	1.17	6.4–7.6	7.0	23–33	28	32–56	44	1.14–1.14	1.14	6.2–6.9	6.6
Wuxian	24	41	1.08	6.6	6–30	23	26–47	37	0.97–1.47	1.22	3.4–7.4	5.6	12–21	17	24–31	28	1.19–1.23	1.21	6.0–7.4	6.7
Wujiang	17	36	1.06	5.9	3–26	15	17–58	38	0.89–1.67	1.28	4.9–6.9	5.9	23–33	28	32–56	44	1.14–1.14	1.14	6.2–6.9	6.6
Wuxi	14	28	1.16	6.7	4–17	11	14–34	24	1.09–1.39	1.24	5.3–7.2	6.3	21–33	27	25–31	28	1.13–1.23	1.18	6.0–6.3	6.1
Jiangyin	13	13	1.28	6.2	6–17	12	8–25	17	0.99–1.54	1.27	5.4–8.0	6.7	21–33	27	25–31	28	1.13–1.23	1.18	6.0–6.3	6.1
Wujin	12	9	1.22	6.8	7–18	13	4–13	9	1.08–1.51	1.30	6.2–7.9	7.1	21–33	27	25–31	28	1.13–1.23	1.18	6.0–6.3	6.1
Jintan	10	9	1.32	6.8	7–14	11	4–13	9	1.17–1.58	1.38	5.5–7.6	6.6	12–21	17	24–31	28	1.19–1.23	1.21	6.0–7.4	6.7
Liyang	10	10	1.23	6.2	6–17	12	7–12	10	1.05–1.37	1.21	6.0–7.2	6.6	12–21	17	24–31	28	1.19–1.23	1.21	6.0–7.4	6.7
Yixing	13	27	1.17	6.0	3–30	17	10–53	32	1.11–1.58	1.35	4.4–8.5	6.5	21–33	27	25–31	28	1.13–1.23	1.18	6.0–6.3	6.1
Dantu	7	36	1.25	6.6	2–19	11	12–49	31	1.07–1.39	1.23	5.8–8.0	6.9	12–21	17	24–31	28	1.19–1.23	1.21	6.0–7.4	6.7
Jurong	10	30	1.23	5.4	6–13	10	15–38	27	1.10–1.29	1.20	5.1–7.4	6.3	12–21	17	24–31	28	1.19–1.23	1.21	6.0–7.4	6.7
Danyang	12	29	1.24	6.7	8–16	12	16–53	35	1.07–1.36	1.22	5.8–7.8	6.8	12–21	17	24–31	28	1.19–1.23	1.21	6.0–7.4	6.7
Jiaxing	19	34	1.19	6.5	10–26	18	20–56	38	0.98–1.34	1.16	5.8–7.6	6.7	23–33	28	32–56	44	1.14–1.14	1.14	6.2–6.9	6.6
Jiashan	21	39	1.23	6.2	15–27	21	22–44	33	1.03–1.34	1.19	5.7–7.0	6.4	23–33	28	32–56	44	1.14–1.14	1.14	6.2–6.9	6.6
Pinghu	15	35	1.10	6.6	9–24	17	22–43	33	0.92–1.48	1.20	6.3–7.2	6.8	11–31	21	18–48	33	1.12–1.27	1.20	5.5–7.4	6.5
Haiyan	17	40	1.17	6.7	7–25	16	22–52	37	0.92–1.51	1.22	5.7–7.3	6.5	11–31	21	18–48	33	1.12–1.27	1.20	5.5–7.4	6.5
Haining	14	36	1.19	6.6	7–24	16	19–52	36	0.92–1.51	1.22	6.0–7.5	6.8	11–31	21	18–48	33	1.12–1.27	1.20	5.5–7.4	6.5
Tongxiang	14	30	1.05	6.5	7–29	18	20–52	36	0.91–1.34	1.13	6.0–7.4	6.7	14–33	24	25–34	30	1.12–1.13	1.13	6.3–6.6	6.5
Huzhou	23	30	1.10	6.2	13–37	25	7–42	25	0.99–1.37	1.18	5.6–6.7	6.2	14–33	24	25–34	30	1.12–1.13	1.13	6.3–6.6	6.5
Changxing	17	31	1.14	5.8	6–31	19	9–47	28	0.84–1.53	1.19	3.6–7.1	5.4	14–33	24	25–34	30	1.12–1.13	1.13	6.3–6.6	6.5
Anji	18	22	1.16	6.0	12–34	23	12–42	27	0.84–1.44	1.14	5.4–6.7	6.1	14–33	24	24–35	30	1.12–1.13	1.13	6.3–6.6	6.5
Deqing	19	32	1.12	6.3	7–26	17	18–38	28	0.87–1.53	1.20	5.2–7.2	6.2	14–33	24	24–35	30	1.12–1.13	1.13	6.3–6.6	6.5
Yuhang	15	5	1.16	6.6	9–21	15	16–48	32	0.95–1.34	1.15	5.9–7.3	6.6	11–31	21	18–48	33	1.12–1.27	1.20	5.5–7.4	6.5
Linan	22	22	1.09	6.2	18–27	23	8–29	19	0.91–1.14	1.03	5.5–7.8	6.7	11–31	21	18–48	33	1.12–1.27	1.20	5.5–7.4	6.5
Minhang	13	26	1.18	7.6	10–18	14	17–46	32	1.11–1.30	1.21	6.4–8.0	7.2	11–31	21	18–48	33	1.12–1.27	1.20	5.5–7.4	6.5
Jiading	13	28	1.10	7.6	9–20	15	13–44	29	0.94–1.24	1.09	6.5–8.1	7.3	11–31	21	18–48	33	1.12–1.27	1.20	5.5–7.4	6.5
Chuangsha	12	29	1.15	7.6	9–20	15	17–36	27	1.06–1.33	1.20	7.3–8.0	7.7	11–31	21	18–48	33	1.12–1.27	1.20	5.5–7.4	6.5
Nanhui	16	31	1.18	7.4	13–22	18	8–35	22	1.11–1.21	1.16	6.5–8.1	7.3	11–31	21	18–48	33	1.12–1.27	1.20	5.5–7.4	6.5
Qingpu	21	27	1.15	7.1	7–33	20	11–36	24	0.94–1.53	1.24	5.6–8.3	7.0	23–33	28	32–56	44	1.14–1.14	1.14	6.2–6.9	6.6
Songjiang	23	26	1.20	6.8	10–33	22	8–37	23	1.03–1.47	1.25	5.6–8.1	6.9	23–33	28	32–56	44	1.14–1.14	1.14	6.2–6.9	6.6
Jinshan	20	29	1.22	7.0	11–37	24	18–36	27	1.11–1.47	1.29	4.6–8.3	6.5	11–31	21	18–48	33	1.12–1.27	1.20	5.5–7.4	6.5
Fengxian	15	25	1.20	7.4	12–18	15	19–39	29	1.11–1.49	1.30	6.9–8.1	7.5	11–31	21	18–48	33	1.12–1.27	1.20	5.5–7.4	6.5
Baoshan	11	23	1.21	7.9	9–19	14	8–44	26	1.11–1.28	1.20	7.2–8.2	7.7	11–31	21	18–48	33	1.12–1.27	1.20	5.5–7.4	6.5
Chongming	10	17	1.11	8.1	9–13	11	15–29	22	1.11–1.21	1.12	7.8–8.1	8.0	12–16	14	24–39	31	1.17–1.27	1.22	7.3–7.4	7.5
**Tai-Lake region**	**15**	**26**	**1.18**	**6.7**	**2**–**38**	**16**	**4**–**58**	**27**	**0.84**–**1.54**	**1.23**	**3.4**–**8.3**	**6.7**	**11**–**33**	**22**	**18**–**56**	**32**	**1.12**–**1.27**	**1.18**	**5.5**–**7.4**	**6.5**

WA = Weighted average of soil properties by the area of each polygon; SOC =  Initial SOC content (g kg^−1^); Clay =  Clay content (%); BD =  Bulk Density (g cm^−3^); Range =  Range of maximum and minimum soil properties; Ave =  Average of maximum and minimum soil properties.

The differentiation of soil properties was also shown at the county scale in the Tai-Lake region (**Table 4**). The average values of initial SOC content and bulk density sourced from C5 for 24 counties were higher than those from P5; the other was that the average values of clay content for 24 counties and pH for 20 counties in C5 were lower than those from P5. Although the average clay content sourced from P5 for 25 counties was slightly lower than that from C14, but the average initial SOC content sourced from C14 for 34 counties was obviously higher than that of P5. According to statistics describing the 1∶50,000 digital soil database of the Tai-Lake region, initial SOC content of six paddy soil subgroups, namely submergenic, bleached, percogenic, hydromorphic, degleyed and gleyed, were 10 g kg^−1^, 10 g kg^−1^, 11 g kg^−1^, 15 g kg^−1^, 19 g kg^−1^, and 25 g kg^−1^, respectively. As map scale decreased from 1∶50,000 to 1∶14,000,000, the submergenic, bleached, percogenic and degleyed subgroups on the 1∶50,000 digital soil map were eliminated and merged into the hydromorphic and degleyed subgroups in the 1∶14,000,000 digital soil map [32], [39]. The initial SOC content of the hydromorphic and gleyed subgroups in the 1∶14,000,000 digital soil database were 17 g kg^−1^ and 28 g kg^−1^, respectively, which were higher than most paddy soil subgroups in the 1∶50,000 digital soil database. Therefore, the average initial SOC content of most counties in C14 was significantly higher than that from P5, while the average values of bulk density for 20 counties and pH for 24 counties in C14 was lower than those from P5. The results demonstrated that the soil properties (i.e., texture, SOC content, bulk density, and pH) in three resolution databases methods had large differences in the Tai-Lake region. Many studies have showed that SOC spatial variability is expressed by map delineations and map unit composition which varied with scales, resulting in the assignment of different soil properties at each scale of aggregation [32], [48], [49]. As such, an improper of soil map scales and simulation unit may lead to SOC estimation inaccuracy.

### Variation of the average annual-, total SOC change modeled with the three resolution databases in Tai-Lake region

**Figure 6:**
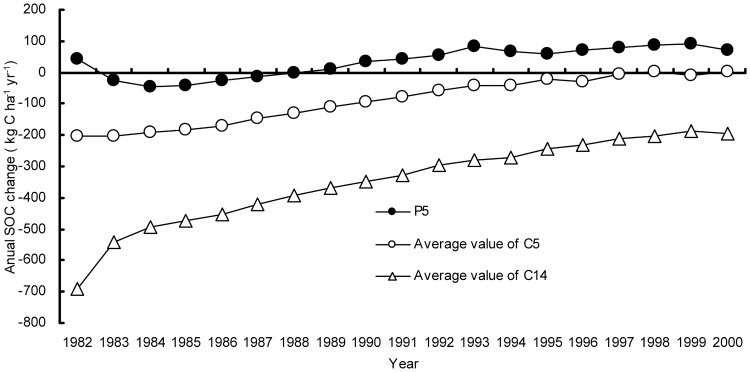
Temporal distribution of average annual SOC change modeled with P5, C5 and C14 from 1982 to 2000 in the Tai-Lake region, China.

Similar trends can be observed in estimates of average annual-, total SOC change over the 19 year study period for three resolution databases decreased from P5 to C14 (**Fig. 6**). Simulation results demonstrate that total SOC change of P5 in the top layer (0–30 cm) of the 2.3 M ha of paddy rice fields in the Tai-Lake region was +1.48 Tg C from 1982 to 2000, with the annual SOC change ranging from -45 kg C ha^−1^ yr^−1^ to 92 kg C ha^−1^ yr^−1^ (**Fig. 6**). From 1982 to 1988, the SOC change modeled with P5 inputs was almost negative with annual changes ranging from -3.2 kg C ha^−1^ yr^−1^ to -45 kg C ha^−1^ yr^−1^. According to agricultural statistical data, chemical fertilizer application rate ranged from 180 kg N ha^−1^ yr^−1^ to 350 kg N ha^−1^ yr^−1^, which is a relatively low value. Low fertilizer application rates often result in reduced SOC sequestration [31], [55]. From 1989 to 2000, rural economic development led to increased fertilizer application from 350 kg N ha^−1^ yr^−1^ to 400 kg N ha^−1^ yr^−1^. Increasing fertilizer application results in enhanced crop production and residue accumulation, and the latter leads to an increase of SOC. Further, much of the region has been utilizing no-tillage practices in planting wheat since 1991, which contribute to reduced SOC decomposition [35].

Although three resolution databases within a certain county have the same feature input value such as crops, agricultural management, and climate; SOC balance of C5 (or C14) in the Tai-Lake region was almost negative with annual changes ranging from 86 kg C ha^−1^ yr^−1^ to -205 kg C ha^−1^ yr^−1^ (or -185 kg C ha^−1^ yr^−1^ to -693 kg C ha^−1^ yr^−1^) from 1982 to 2000 (**Fig. 6**). The total SOC changes of C5 and C14 in the Tai-Lake region were −3.99 Tg C and −15.38 Tg C, respectively, from 1982 to 2000. With the total SOC change as modeled with P5 inputs as the baseline, the relative deviation of C5 and C14 were 368% and 1126%, respectively.

**Figure 7:**
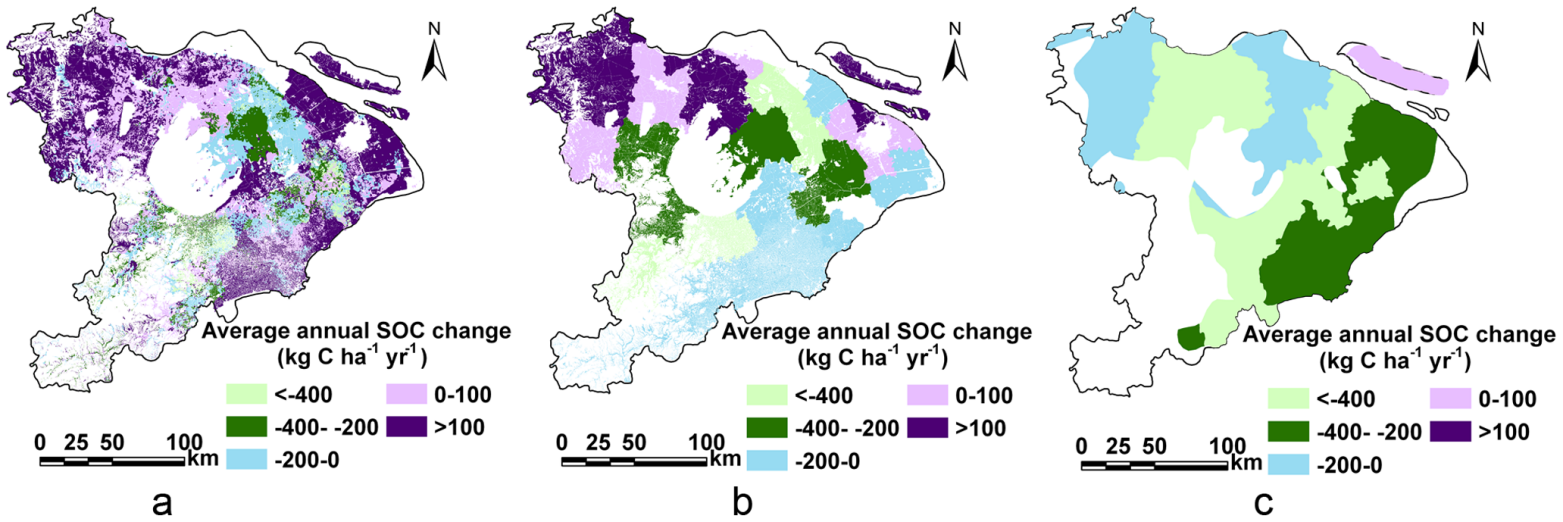
Spatial distribution of average annual SOC change modeled with P5, C5 and C14 in the Tai-Lake region, China (a: P5, b: c5, and c:C14).

As Table 3 illustrated, initial SOC content was the most sensitive parameter controlling SOC change among all soil factors in P5 and C5 [20], [22]. The average initial SOC value of P5 and C5 were 15 g kg^−1^ and 16 g kg^−1^ for the entire Tai-Lake region, respectively. Furthermore, the average initial SOC content sourced from P5 for 24 counties was lower than that from C5, while the average clay content sourced from P5 for 24 counties was also higher than that from C5. Many previous studies showed that soils with lower initial organic carbon and higher clay content tended to sequester C [20], [22], [35]. The high SOC sequestration rate (34 kg C ha^−1^ yr ^−1^) was thus associated with P5 (**Fig. 7a**). Conversely, the high SOC losses rate (-91 kg C ha^−1^ yr^−1^) was associated with C5 (**Fig. 7b**). The SOC losses rate (-349 kg C ha^−1^ yr^−1^) in C14 was the highest in the three resolution databases (**Fig. 7c**). Table 3 demonstrates that pH and initial SOC content are the most sensitive parameters controlling SOC change among all soil factors in C14. The average initial SOC value (22 g kg^−1^) of C14 was significantly higher than that of P5 (15 g kg^−1^) and C5 (16 g kg^−1^) for the entire Tai-Lake region. In addition, the average pH value of C14 for 34 counties was close to neutral (6.5–7.5), and the average initial SOC contents of C14 for 28 counties were higher than 20 g kg^−1^. Some studies showed that soils with neutral pH value and higher organic carbon content were favorable for CO_2_ production by providing more substrates and better living environment for microbes [22], [56].

The comparison illustrates that using different basic simulation units and soil data sources will produce different conclusions as to C sequestration or C liberation in the same study area. The implication is that more precise soil data and high resolution simulation units were necessary for better simulating regional scale SOC dynamics. The simulation outcome can be attributed to how the databases represent soil types and spatial heterogeneity, which is more precisely done with larger scale soil data and high resolution simulation units (e.g., 1∶50,000 soil database).

### Distribution of the average annual-, total SOC change modeled with the three resolution databases in different counties

The differentiation of the average annual-, total SOC change in P5, C5 and C14 was also shown at the county scale in the Tai-Lake region (**Table 5** **and** **Fig. 7**). In the modeled domain, there were 26 counties that gained SOC and 11 counties that lost SOC from 1982 to 2000 in P5. The highest SOC sequestration rate of P5 were in Dantu, Jurong, Jiading and Baoshan counties which was higher than 200 kg C ha^−1^ yr^−1^, due to the low initial SOC content (7.1 g kg^−1^, 9.5 g kg^−1^, 13 g kg^−1^ and 11 g kg^−1^, respectively). In addition, the clay content of P5 in Dantu and Jurong counties were 36% and 30%, respectively. High clay content is associated with high SOC sequestration [22], [57], [58]. By contrast, the greatest SOC loss rate of P5 in the Huzhou, Songjiang, Linan and Wuxian county was more than 170 kg C ha^−1^ yr^−1^, due to the high initial SOC content (23 g kg^−1^, 23 g kg^−1^, 22 g kg^−1^ and 24 g kg^−1^, respectively). Moreover, the clay content of P5 in Linan and Songjiang counties were only 22% and 26%, respectively. Low clay content is linked to high CO_2_ emissions [22].

**Table 5:** Distribution of the average annual SOC change (kg C ha^−1^ yr^−1^) and the total SOC change (Gg C) in different counties of the Tai-Lake region, China modeled with P5, C5 and C14 from 1982 to 2000.

County	Area 10^4^ha	P5		C5						C14					
		ASC	TSC	ASC			TSC			ASC			TSC		
		WA		Max	Min	AVE	Max	Min	AVE	Max	Min	AVE	Max	Min	AVE
Zhangjiagang	2.54	2	1	138	−50	44	66	−24	21	57	−418	−181	27	−202	−87
Changshu	7.55	−64	−92	246	−1438	−596	353	−2063	−855	92	−404	−156	132	−580	−224
Taicnang	6.14	43	50	241	−360	−60	281	−420	−70	192	−993	−400	224	−1158	−467
Kunshan	7.57	38	55	252	−1057	−402	362	−1520	−579	−75	−838	−456	−108	−1205	−656
Wuxian	14.78	−172	−483	356	−876	−260	998	−2458	−730	72	−403	−166	201	−1132	−466
Wujiang	9.79	53	99	586	−717	−65	1091	−1333	−121	−59	−792	−425	−110	−1472	−791
Wuxi	9.77	49	91	478	−196	141	888	−364	262	−256	−958	−607	−476	−1778	−1127
Jiangyin	8.69	2	4	313	−101	106	517	−167	175	−262	−951	−606	−432	−1569	−1000
Wujin	14.85	91	256	248	−57	96	701	−160	270	−274	−969	−621	−774	−2734	−1754
Jintan	7.10	146	197	245	81	163	331	110	220	136	−364	−114	184	−492	−154
Liyang	10.84	177	365	302	−115	94	623	−237	193	127	−386	−129	263	−796	−267
Yixing	10.34	147	289	514	−1011	−248	1011	−1987	−488	−262	−950	−606	−514	−1867	−1191
Dantu	5.07	371	357	615	−276	170	592	−266	163	141	−367	−113	136	−353	−109
Jurong	8.03	238	363	403	−8	198	614	−12	301	125	−374	−124	191	−570	−190
Danyang	9.58	47	85	416	−152	132	758	−276	241	130	−364	−117	237	−663	−213
Jiaxing	6.57	−124	−155	412	−523	−55	514	−652	−69	−11	−752	−381	−13	−938	−476
Jiashan	4.13	−103	−81	110	−631	−260	87	−494	−204	−48	−786	−417	−38	−616	−327
Pinghu	4.81	127	116	349	−466	−58	319	−425	−53	274	−923	−324	250	−843	−296
Haiyan	2.74	41	21	489	−549	−30	254	−285	−16	279	−943	−332	145	−490	−173
Haining	3.92	151	113	483	−556	−36	359	−413	−27	274	−951	−338	204	−708	−252
Tongxiang	4.42	128	107	504	−634	−65	424	−533	−55	122	−846	−362	102	−711	−304
Huzhou	6.02	−309	−353	163	−1184	−511	186	−1354	−584	92	−915	−411	106	−1046	−470
Changxing	5.62	42	45	422	−863	−221	450	−922	−236	71	−901	−415	76	−962	−443
Anji	4.18	−66	−52	178	−1025	−423	141	−814	−336	58	−873	−408	46	−694	−324
Deqing	3.11	17	10	379	−684	−152	224	−404	−90	77	−930	−426	46	−550	−252
Yuhang	5.27	−91	−92	344	−371	−13	345	−372	−13	120	−1061	−456	120	−1034	−457
Linan	3.06	−220	−128	7	−381	−187	4	−222	−109	218	−754	−268	127	−439	−156
Minhang	3.49	62	41	294	−239	28	195	−158	18	257	−974	−359	171	−645	−238
Jiading	4.29	238	194	387	−197	95	315	−161	77	281	−940	−329	229	−766	−268
Chuangsha	3.71	188	133	339	−295	22	239	−208	15	286	−925	−319	202	−653	−225
Nanhui	4.11	152	119	189	−279	−45	148	−208	−35	287	−939	−326	224	−734	−255
Qingpu	5.68	−52	−56	432	−1042	−305	467	−1125	−329	2	−756	−377	3	−816	−407
Songjiang	5.90	−287	−322	249	−1010	−381	278	−1131	−426	−60	−816	−438	−67	−914	−491
Jinshan	5.63	−77	−83	213	−1481	−634	228	−1585	−679	275	−945	−335	294	−1011	−359
Fengxian	5.87	9	10	171	−270	−49	191	−301	−55	280	−944	−332	321	−1053	−370
Baoshan	3.13	200	119	393	−92	151	234	−55	90	311	−876	−283	185	−522	−168
Chongming	3.73	195	138	283	47	165	201	34	117	165	−108	28	117	−77	20
**Tai-Lake region**	**232**	**34**	**1483**	**340**	−**521**	−**91**	**14987**	−**22977**	−**3994**	**46**	−**744**	−**349**	**2022**	−**32790**	−**15380**

WA =  Weighted average of annual mean SOC change (kg C ha^−1^ yr^−1^) by the area of each polygon; ASC =  Average annual SOC change (kg C ha^−1^ yr^−1^); TSC =  Total SOC change (Gg C); Max =  Maximum value of ASC (or TSC); Min =  Minimum value of ASC (or TSC); Ave =  Average of maximum and minimum ASC (or TSC).

However, under the same agricultural practice, there were only 14 counties that gained SOC and 23 counties that lost SOC from 1982 to 2000 in C5. The highest SOC sequestration rate of C5 were in Dantu, Jurong, Jintan, Chongming, and Baoshan counties which was higher than 150 kg C ha^−1^ yr^−1^. The main reason was that the initial SOC content of C5 in Dantu, Jurong, Jintan, Chongming, and Baoshan counties were 11 g kg^−1^, 10 g kg^−1^, 11 g kg^−1^, 11 g kg^−1^ and 14 g kg^−1^, respectively; the other was that the average clay content of C5 in Dantu, Jurong, and Baoshan counties ranged from 26% to 31%. Some studies showed that low initial SOC value and high clay content were linked to low CO_2_ emissions [22], [57], [58]. In contrast, the greatest SOC loss rate of C5 in Jinshan, Changshu, Huzhou, Anji, and Kunshan county were more than 400 kg C ha^−1^ yr^−1^, which possessed high initial SOC and low bulk density [22], [35]. Compared with the P5 resolution database, the average annual-, total SOC change modeled with C5 for 28 counties was lower than that from P5. With the total SOC change as modeled with P5 inputs as the baseline, the relative deviations of counties in Jiangyin, Zhangjiagang and Kunshan were relatively high (>1000%). The relative deviations ranged from 50% to 250% in most counties. Only fifteen counties (Wuxian, Wujin, Jintan, Liyang, Dantu, Jurong, Huzhou, Yuhang, Linan, Minhang, Jiading, Chuangsha, Songjiang, Baoshan, and Chongming) had relatively low value of relative deviation (<100%). The SOC changes for the two resolution databases are almost in agreement with the soil feature across the 37 simulated counties (**Table 4** **and** **Table 5**). The average initial SOC content sourced from C5 for 24 counties was higher than that from P5, and the average clay content sourced from C5 for 24 counties was also lower than that from P5. Some research showed that high initial SOC content and low clay content is favorable for C losses [22], [57], [58].

As can be seen from the Table 5, a big number of counties where the average annual-, total SOC change modeled with the C14 and P5 differed greatly. There was only one county that gained SOC from 1982 to 2000, while other 36 counties lost SOC in C14. The SOC losses of C14 ranged from 360 kg C ha^−1^ yr^−1^ to 620 kg C ha^−1^ yr^−1^ in most counties. With the total SOC change as modeled with P5 inputs as the baseline, the relative deviations of counties in Zhangjiagang, Taicang, Kunshan, Wuxi, Jiangyin, Changxing, Deqing, and Fengxian were more than 1000%. Only five counties (Wuxian, Huzhou, Linan, Songjiang, and Chongming) in C14 had relatively low deviation (<100%). The main reasons were that the average pH value of C14 in most counties ranged from 6.5 to 7.5, which were closer to neutral than that from C5 and C14. Moreover, the average initial SOC contents of C14 in most counties were higher than 20 g kg^−1^, which was also much higher than that from P5 or C5. Therefore, high SOC losses occurred in C14.

The modeled data at county scale in three simulation methods indicated the underestimation with the county-based database was related to its soil data source and simulation unit resolution, especially the coarse soil maps (1∶14,000,000) that missed relatively small soil patches containing low or high soil properties (i.e., initial SOC content, pH, and clay content) which were sensitive to SOC change. This would also explain why the precision of soil database plays an important role in elevating the accuracy of modeled SOC change at regional scale.

## Conclusions

Using different spatial information, process-based models integrated with GIS databases can play an important role in describing C biogeochemical cycles, such as targeting mitigation efforts to the most beneficial regions. However, SOC models have often been applied to regions with high heterogeneity but limited spatially differentiated soil information and simulated unit resolution.

Simulation results indicate that total SOC change from 1982 to 2000 in the top layer (0–30 cm) of the 2.3 M ha of paddy rice fields in the Tai-Lake region was +1.48 Tg C for P5. However, discrepancies in the results existed among the three databases, because different soil data and basic simulation units were used. The total SOC changes in the Tai-Lake region were -3.99 Tg C and -15.38 Tg C for C5 (or C14), respectively, from 1982 to 2000. With the total SOC change as modeled with P5 inputs as the baseline, the relative deviation of C5 was lower than C14 due to the more precise soil data. In contrast, the relative deviation of C14 was higher than other databases due to using coarser soil data and low-resolution simulation units. In addition, with the same basic simulation unit, average annual-, total SOC change between C5 and C14 for the Tai-Lake region also had a large discrepancy due to the use of different soil data. The comparison demonstrated that the most sensitive factors (e.g., initial SOC content and pH) for modeling SOC dynamics should be given a high priority during the input data acquisition as they contribute disproportionately to the uncertainties produced during the upscaling process [20]. The results also indicate that improving the performance of the biogeochemical DNDC model is essential in creating accurate models of the soil carbon cycle.
